# UCA1 Overexpression Promotes Hypoxic Breast Cancer Cell Proliferation and Inhibits Apoptosis via HIF-1*α* Activation

**DOI:** 10.1155/2021/5512156

**Published:** 2021-05-07

**Authors:** Hani Choudhry

**Affiliations:** Department of Biochemistry, Center for Artificial Intelligence in Precision Medicines, King Abdulaziz University, Jeddah, Saudi Arabia

## Abstract

The noncoding RNA termed urothelial carcinoma-associated 1 (UCA1) is an oncogenic lncRNA involved in promoting the growth of several tumors through various pathways. The aim of this study was to explore the expression of UCA1 in hypoxic breast cancer and its impact on tumorigenesis in low levels of oxygen. Here, we show that UCA1 is upregulated in a number of hypoxic (1% O_2_) breast cancer cells. In addition, UCA1 expression is significantly overexpressed in breast cancer tissues compared to matched normal cells. UCA1 knockdown in hypoxia inhibits breast cancer proliferation and induces apoptosis. The knockdown of hypoxia-inducible transcription factor 1*α* (HIF-1*α*) but not HIF-2*α* significantly decreases the expression of UCA1 in hypoxia. Overall, these findings indicate that UCA1 is a hallmark of hypoxic breast cancer and its expression is positively regulated by HIF-1*α*.

## 1. Introduction

Breast cancer is considered to be one of the most common solid tumors to involve a hypoxic tumor microenvironment and resist treatment [1–3]. Cancer cells have adapted different mechanisms to resist radiotherapy and chemotherapy, including silencing of tumor suppressor genes and activation of various oncogenes, which leads to tumor growth and metastasis [4–7]. The hypoxic tumor microenvironment is considered to be one of the important factors in overcoming treatment of solid tumors, including breast cancer [1, 8–10]. Activation of hypoxia-inducible transcription factor (HIF-1) is used by cancer cells to adapt to the hypoxic microenvironment through regulating the expression of several genes involved in various biological process, such as cell proliferation, apoptosis, and tumor metastasis [11–13]. HIF is a heterodimer consisting of two subunits, HIF-1*α*, which is O_2_-sensitive, and HIF-1*β*, a ubiquitous expressed subunit [14]. Activated HIF-1*α* translocates to the nucleus and binds to the promoters of its target genes, leading to their activation [15, 16], with subsequent enhancement of cell proliferation and metastasis and inhibition of apoptosis [17–22].

Several studies have shown that HIF-1*α* regulates the expression of protein-coding genes in breast cancer [23–27]. Considering the fact that the majority of breast cancer cell deaths can be attributed to metastasis [28], understanding the key regulators involved in the promotion of breast cancer metastasis under stress conditions will be helpful in finding new targets for this cancer.

Beside its role in the regulation of protein-coding genes, HIF-1*α* has been shown to regulate several long noncoding RNAs (lncRNAs) [11, 29–33]. LncRNAs are regulatory RNAs with a length of >200 nucleotides [34]. LncRNA transcripts are involved in cancer biology through their interactions with several targets, including DNA, RNA, and protein [31]. They are involved in modulating gene transcription, mRNA stability and splicing, and other epigenetic processes, leading to the regulation of many biological processes, such as apoptosis, cell cycle proliferation, and autophagy [35]. Several works have shown that lncRNAs are deregulated in various types of cancer, including breast cancer, leading to tumor progression and metastasis [36–41]. The noncoding RNA urothelial carcinoma-associated 1 (lncRNA-UCA) is an oncogene able to promote the development and proliferation of breast cancer cells through its effects on several pathways, including inhibition of p27 (Kip1) [42] and p21 [43], inhibition of tumor suppressors miR-143 [44] and miR-206 [45] and the Wnt/*β*-catenin pathway [46], regulation of miR-18a [47], regulation of phosphatidylinositol 3 kinase (PI3K)/protein kinase B (AKT) signaling pathway [43], and inhibition of mTOR (mechanistic target of rapamycin) signaling pathway [48]. Although the role of lncRNA-UCA in cancer growth has been documented, the signaling pathways underlying UCA1 in hypoxic breast cancer are poorly elucidated.

The aim of this study was to investigate the expression of lncRNA-UCA in various breast cancer cell lines under hypoxic conditions and elucidate the role of UCA1 in tumorigenesis. We show that UCA1 is commonly upregulated in hypoxic breast cancer cells and its overexpression is associated with tumor growth and inhibition of apoptosis in an HIF-dependent manner. UCA1 knockdown induces inhibition of breast cancer cell proliferation and apoptosis under hypoxic conditions.

## 2. Materials and Methods

### 2.1. Cell Culture and Treatment

Cells of human breast cancer cell lines MCF-7, SKBR3, MDA-MB-468, MDA-MB-231, BT474, BT-20, and T47D were obtained from the American Type Culture Collection (ATCC, Manassas, VA, USA). Cell lines were maintained in a humidified incubator with 5% CO_2_ at 37°C and grown in DMEM (Lonza, Switzerland) supplemented with 10% heat-inactivated fetal bovine serum (FBS). All media were supplemented with 2 mM L-glutamine, 100 *µ*g/mL gentamycin, 100 U/mL penicillin, and 50 mg/mL streptomycin (Sigma, St. Louis, MO, USA). Cells were incubated for 24 h in a hypoxia workstation (Ruskinn Technology Ltd., Bridgend, UK).

### 2.2. Cell Proliferation Assay

MCF-7 cells were seeded, in triplicate, on 96-well plates at a density of 10^3^ cells/well for 24 h. Cell proliferation was measured using the CyQUANT NF Cell Proliferation Assay Kit (Invitrogen-Life Technologies, USA) in cells that were transfected with human UCA1 sequences or scrambled control (Integrated DNA Technologies, Coralville, IA, USA) as described in Section 2.4.

### 2.3. Annexin V Apoptosis Assay

MCF-7 cells were seeded in triplicate on 6-well plates and cultured until a confluency of 60% was reached. Cells that were transfected with human UCA1 sequences or scrambled control (Section 2.4) were collected, stained for annexin V (Life Technologies, USA), counterstained with propidium iodide, and analyzed using a CyAn ADP FACS analyzer (Beckman Coulter, High Wycombe, UK).

### 2.4. siRNA Transfection

MCF-7 cells were plated in 6-well plates and cultured until a confluency of 60% was reached. Cells were transfected with human HIF-1*α* siRNA, HIF-2*α* siRNA, or UCA1 siRNA sequences or scrambled control siRNA (Integrated DNA Technologies, Coralville, IA, USA). Transfections were performed using lipofectamine RNAiMAX reagent (Invitrogen, Life Technologies) following the manufacturer's instructions (Table 1).

### 2.5. Real-Time PCR Analysis

The mirVana miRNA Isolation Kit (Ambion, Life Technologies, Paisley, UK) was used for the isolation of total RNA from cell lines and tissues. All the isolated RNAs were treated with DNase I (TURBO DNA-free, Ambion, Life Technologies). SuperScript II reverse transcriptase (Invitrogen, Life Technologies, Paisley, UK) was used to synthesize complementary DNA (cDNA). Real-time PCR was performed using IQ SYBR Green Mix (Bio-Rad, Hemel Hempstead, UK) on the CFX96 Real-Time System (Bio-Rad) and with normalization to 60S ribosomal protein L11 (RPL11). Primer sequences used for qPCR assays are given (Table 2).

### 2.6. Analysis of Tumors

UCA1 expression analysis was conducted on matched tumors and healthy (*n* = 25) biopsies from women with breast cancer who had not previously received any treatment at King Abdulaziz University Hospital (KAUH), Jeddah, Saudi Arabia. The Research Committee of the Unit of Biomedical Ethics approved this study, and all patients signed the consent form. Patient information was obtained through a standard questionnaire, and the anthropometric data were collected using standard and well-established methods. Total RNA was isolated from samples and subjected to qPCR to measure the UCA1 expression levels. The Research Committee of the Unit of Biomedical Ethics at King Abdulaziz University Hospital (KAUH), Jeddah, Saudi Arabia, approved this study. The informed consent was obtained from all subjects.

### 2.7. Statistical Analysis

Statistical analyses were performed in R (http://www.R-project.org) using two-tailed t-tests or one-way analysis of variance with Dunnett's or Bonferroni posttest as appropriate. The 2^−ΔΔCt^ method was used for gene expression analysis. All experiments were performed using 3 biological replicates. All methods were carried out in accordance with relevant guidelines and regulations.

## 3. Results

### 3.1. UCA1 Is Significantly Induced in Hypoxic Condition in MCF-7 Cells

**Figure 1:**
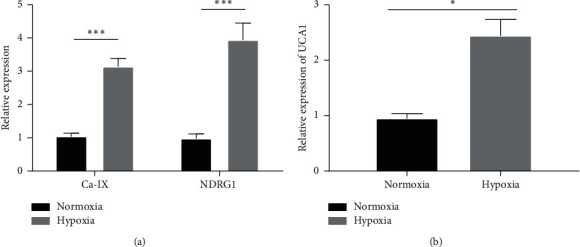
(a) Upregulation of hypoxia-induced genes (Ca-IX and NDRG1) in MCF-7 cells (1% O2 for 24 h confirms induction of hypoxia). ^*∗*^*p* < 0.01; ^*∗∗∗*^*p* < 0.0001. (b) Upregulation of UCA1 lncRNA in hypoxic MCF-7 cells (1% O_2_ for 24 h).

To evaluate the expression of UCA1, we grew MCF-7 cells in normoxic (21% oxygen) and hypoxic (1% oxygen) conditions. Carbonic anhydrase IX (Ca-IX) and N-Myc downregulated gene 1 (NDRG1) are known as key regulators of cell hypoxia that promote tumor cell survival [49]. To confirm the induction of hypoxia in the MCF-7 breast cancer cell line, the expression of Ca-IX and NDRG1 in MCF-7 was investigated in normoxic and hypoxic conditions. As shown in Figure 1(a), the expression of both Ca-IX and NDRG1 significantly increased in MCF-7 cells under hypoxic compared to normoxic conditions. A >3-fold increase in Ca-IX (fold change (FC) = 3.13, *p* < 0.0001) and 3-fold increase in NDRG1 (FC = 3.93, *p* < 0.0001) expression levels were found under hypoxia (Figure 1(a)). The expression of UCA1 in MCF-7 at the transcriptional level was then analyzed. The expression of UCA1 was significantly increased in MCF-7 cells in hypoxic compared to normoxic conditions (Figure 1(b)). A 2.1-fold increase (*p*=0.002) in UCA1 was found under hypoxic conditions (Figure 1(b)). These data confirm that the MCF-7 breast cancer cell line overexpresses UCA1 in response to hypoxia.

### 3.2. Hypoxia Increases Transcriptional Expression of UCA1 in MCF-7 Cells Most Likely through Activation of HIF-1*α*

**Figure 2:**
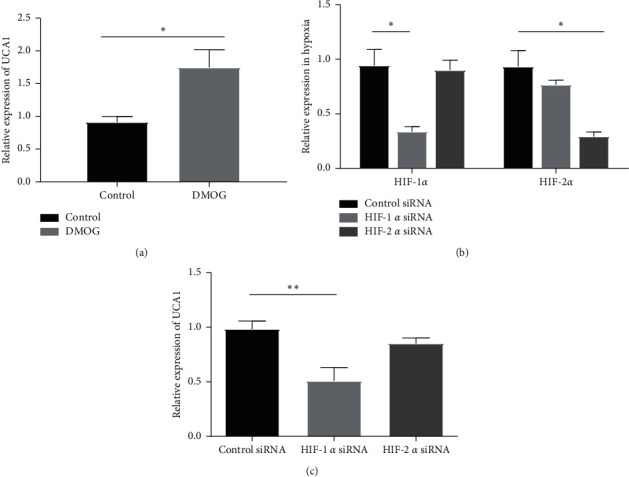
(a) Significant upregulation of UCA1 lncRNA in MCF-7 cells with DMOG treatment suggests UCA1 is regulated by HIF. (b) Effective HIF-1*α* and HIF-2*α* knockdown in hypoxic MCF-7 cells using HIF-1*α* and HIF-2*α* siRNA, respectively. (c) Significant downregulation of UCA1 lncRNA upon HIF-1a siRNA in hypoxic MCF-7 cells, suggesting that UCA1 expression is HIF-1a-dependent in hypoxia. HIF-2a siRNA did not affect expression of UCA1. ^*∗*^*p* < 0.02; ^*∗∗*^*p* < 0.005.

HIF-1*α* is a major transcription factor that regulates hypoxia-regulated genes [50]. Thus, we wanted to investigate whether the increased UCA1 expression in MCF-7 cells (Figure 1(b)) is HIF-dependent. To study this hypothesis, we used dimethyloxalylglycine (DMOG), an inhibitor of HIF-1*α* degradation. As shown in Figure 2(a), treating MCF-7 cells with DMOG significantly increased the expression of UCA1 (FC = 1.78, *p*=0.02), suggesting that HIF-1*α* activates the expression of UCA1 in breast cancer cells under hypoxic conditions. To directly address the role of HIF-1*α* in the regulation of UCA1 expression, knockdown of HIF-1*α* and HIF-2*α* was performed in MCF-7 cells (Figure 2(b)). Depletion of HIF-1*α* and HIF-2*α* resulted in significant depletion of their expression up to 70% in MCF-7 cells (Figure 2(b)). Interestingly, HIF-1*α* knockdown UCA1 (FC = −2, *p*=0.005) but not HIF-2*α* was significantly downregulated in hypoxic MCF-7 cells (Figure 2(c)). These findings show that HIF-1*α* depletion decreases UCA1 expression in breast cancer, indicating a key role of HIF-1*α* in the regulation of UCA1 expression in hypoxic MCF-7 breast cancer cells and, thereby, in tumor growth and survival.

### 3.3. UCA1 Is Upregulated in Several Breast Cancer Cell Lines and Breast Tumors Compared to Matched Normal Tissues

**Figure 3:**
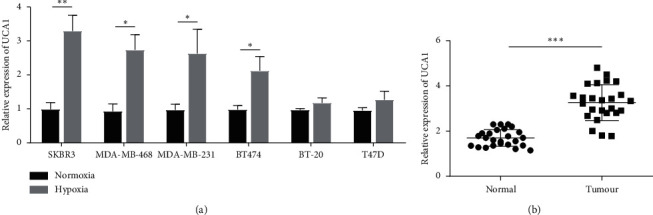
(a) Expression of UCA1 lncRNA in hypoxic breast cancer cells (1% O_2_, 24 h). (b) High expression of UCA1 in breast cancer tumors compared to matched normal tissues. ^*∗*^*p* < 0.05; ^*∗∗*^*p* < 0.001; ^*∗∗∗*^*p* < 0.0001.

To determine whether UCA1 overexpression is a hallmark of hypoxic breast cancer cells, the expression of UCA1 was investigated in several breast cancer cell lines under hypoxic conditions.  Figure 3(a) shows that UCA1 was significantly upregulated in SKBR3 (FC = 3.29, *p*=0.01), MDA-MB-468 (FC = 2.74, *p*=0.02), MDA-MB-231 (FC = 2.63, *p*=0.05), and BT474 (FC = 2.12, *p*=0.04) breast cancer cell lines. A slight increasing trend in UCA1 expression was observed in BT-20 and T47D cell lines (Figure 3(a)). Next, we evaluated the expression of UCA1 in primary breast cancer tissues. Total RNA was isolated from 25 primary tumor-matched normal tissues. High UCA1 expression levels (FC = 3.37, *p* < 0.0001) were found in breast tumors compared to matched normal tissues (Figure 3(b)). These data suggest that the increased migration and invasion of hypoxic breast cancer can be attributed, in large part, to the high expression of UCA1 in these tumors and also indicate that UCA1 could be a diagnostic marker of breast cancer. However, further investigation is needed to confirm this.

### 3.4. UCA1 Silencing Induces Cell Proliferation Inhibition and Apoptosis of Hypoxic Breast Cancer Cells

**Figure 4:**
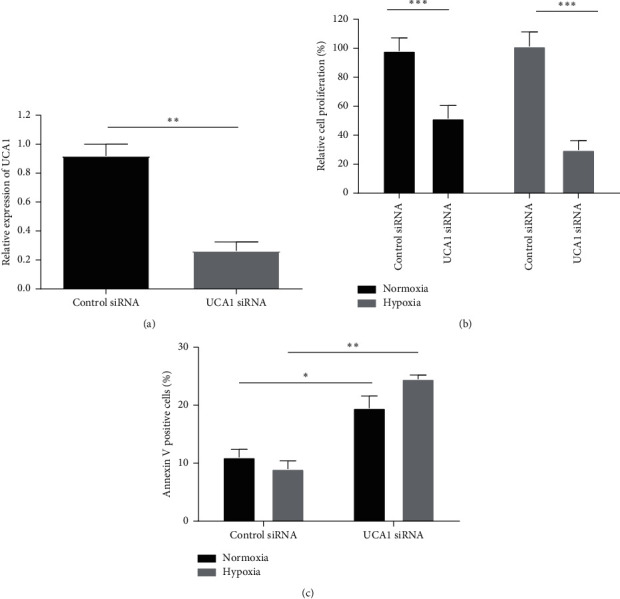
(a) Effective knockdown of UCA1 expression by siRNA in hypoxic MCF-7 cells. (b) Significant decrease of cell proliferation upon UCA1 knockdown by siRNA in normoxic and hypoxic MCF-7 cells. (c) Significant increase in apoptosis upon UCA1 knockdown by siRNA in normoxic and hypoxic MCF-7 cells.

To investigate the cellular function of UCA1 in breast cancer cells under hypoxic conditions, we transfected MCF-7 using siRNAs against UCA1, achieving 87% downregulation of UCA1 expression (Figure 4(a)). The inhibition of UCA1 expression by UCA1 siRNAs caused a significant decrease in MCF-7 cell viability under both normoxic and hypoxic conditions (FC = 0.26, *p*=0.0003) relative to scramble control UCA1-transfected MCF-7 (Figure 4(a)). Then, the functional role of UCA1 silencing on apoptosis was evaluated (Figure 4(b)). UCA1 depletion induced a significant increase in annexin V-positive MCF-7 cells compared to negative UCA1-transfected MCF-7 cells under normoxic and hypoxic conditions (FC = 19.5, *p*=0.04 and FC = 24.5, *p*=0.005, respectively) (Figure 4(c)). Taken together, these findings suggest that HIF-1*α*-induced UCA1 overexpression is involved in the promotion of cell viability and inhibition of apoptosis in hypoxic breast cancer cells.

## 4. Discussion

Malignant cells adapt to hypoxia to promote their growth and metastasis in addition to their ability to resist therapy. This process involves the stabilization of HIF-1*α*, which acts as a transcription factor for several oncogenes in cancer cells, including breast cancer. UCA1 is one of the HIF-1*α*-regulated lncRNAs in hypoxic breast cancer. However, the role of HIF-1*α* in the regulation of UCA1 in breast cancer has rarely been investigated.

In the present study, we first studied the expression of UCA1 in the MCF-7 breast cancer cell line under both normoxic and hypoxic conditions. We showed that, under hypoxic conditions, the expression of UCA1 was increased in MCF-7 breast cancer cells, and this overexpression was associated with significantly increased expression of Ca-IX and NDRG1, which are known as markers of hypoxia. UCA1 knockdown in MCF-7 inhibited cell proliferation and induced apoptosis under both normoxic and hypoxic conditions, with a slightly higher number of apoptotic cells in the latter. These findings are in accordance with previous studies showing that UCA1 depletion inhibited cell proliferation, migration, and invasion and increased apoptosis in normal [51] and hypoxic [21] bladder cancer cells.

HIF-1*α* was shown to regulate the expression of UCA1 in bladder cancer cells by binding to two hypoxia responsive elements (HREs) in the promoter of UCA1, leading to its activation under hypoxic conditions [21]. Similar to these observations, our study shows that knockdown of HIF-1*α*, but not HIF-2*α*, in breast cancer cells induced a significant decrease in UCA1 expression, suggesting that HIF-1*α* is a direct regulator of UCA1 in MCF-7 under hypoxic conditions. Under normoxic conditions, the HIF-1*α* subunit is hydroxylated by HIF-1*α* prolyl hydroxylase, inducing its ubiquitination and subsequent degradation in the proteasome [52]. In response to hypoxic conditions, HIF-1*α* is stabilized and translocated to the nucleus, leading to the activation of several oncogenes, including UCA1 [21]. It is well known that the cell-permeable competitive inhibitor of HIF-1*α* prolyl hydroxylase, DMOG, stabilizes HIF-1*α*, leading to the activation of HIF-1*α*-regulated genes [53]. Our results show that exposure of MCF-7 cells to DMOG significantly increased the expression of UCA1, providing additional evidence that UCA1 expression in breast cancer is under the control of HIF-1*α*.

UCA1 has been shown to be overexpressed in several breast cancer cells, including MCF-7 [48, 54, 55], LM2-4 [56], and MDA-MB-231 [57, 58]. UCA1 was also reported to be upregulated in breast cancer compared to matched normal tissues [44]. Our study shows that, under hypoxic conditions, UCA1 is overexpressed in several breast cancer cells—SKBR3, MDA-MB-468, MDA-MB-231, and BT474—compared with normoxic conditions. Our data also show high expression levels of UCA1 in tumor tissues compared to matched normal tissues. These findings indicate that UCA1 could be a prognostic and diagnostic marker for a hypoxic microenvironment in breast and other cancers; however, further evaluation is needed for confirmation.

## 5. Conclusions

The current study is the first to reveal that UCA1 is upregulated in hypoxic breast cancer cells and tumor tissues, promoting cell proliferation and inhibiting apoptosis. These findings suggest that lncRNA-UCA1 overexpression is orchestrated by the transcription factor HIF-1*α* in hypoxic breast cancer and that targeting this pathway could be a promising strategy for cancer therapy.

## Figures and Tables

**Table 1:** List of siRNA target sequences.

Name	siRNA sequences
Control siRNA	5'-GGUGCGUUGAAGGUAUCA-3'; 5'-UCUGCUGAGUAUGU-3'
UCA1 siRNA	5'-GCGUGGUGUAUUGAGGGCAUCA-3'; 5'-UCUGACUGAUUGGAAGGU-3'
HIF-1*α* siRNA	5'-CUGAUGACCAGCAACUUGAtt-3'; 5'-UCAAGUUGCUGGUCAUCAGtt-3'
HIF-2*α* siRNA	5'-CAGCAUCUUUGAUAGCAGUtt-3'; 5'-ACUGCUAUCAAAGAUGCUGtt-3'

**Table 2:** List of primers used in qPCR.

Gene symbol	Forward primer	Reverse primer
UCA1	5'-ACGCTAACTGGCACCTTGTT-3'	5'-CTCCGGACTGCTTCAAGTGT-3'
NDRG1	5'-CTCTGTTCACGTCACGCTGT-3'	5'-CTCCACCATCTCAGGGTTGT-3'
Ca-IX	5'-ACAATGTGCATATCGTGGCA-3'	5'-CAGAGGGGCTCTGATAGGTTT-3'
HIF-1*α*	5'-TTACAGCAGCCAGACGATCA-3'	5'-CCCTGCAGTAGGTTTCTGCT-3'
HIF-2*α*	5'-TGGGATCTAACAGGAACAGC-3'	5'-CTAAATAGCCAGACAAGGGT-3'
RPL11	5'-CTTTGGCATCCGGAGAAAT-3'	5'-TCCAAGATTTCTTCTGCCTTG-3'

## Data Availability

The data used to support the findings of this study are included within the article.
